# The relationship between liver enzymes, prehypertension and hypertension in the Azar cohort population

**DOI:** 10.1186/s12872-024-03969-x

**Published:** 2024-06-07

**Authors:** Mohammd hossein Somi, Elnaz Faramarzi, Sima Jahangiry, Sarvin Sanaie, Roghayeh Molani-Gol

**Affiliations:** 1 Liver and Gastrointestinal Diseases Research Center of Tabriz university of medical sciences, Tabriz, Iran; 2 Liver and Gastrointestinal Diseases Research Center, Tabriz University of Medical Sciences, Tabriz, Iran; 3 Student Research Committee, Tabriz University of Medical Sciences, Tabriz, Iran; 4 Research center for integrative Medicine in Aging, Aging Research Institute, Tabriz University of Medical Sciences, Tabriz, Iran; 5 Student Research Committee, Tabriz University of Medical Sciences, Tabriz, Iran

**Keywords:** Liver enzymes, Prehypertension, Hypertension

## Abstract

**Background:**

The incidence of hypertension (HTN) as a worldwide health problem is rising rapidly. Early identification and management of pre-HTN before HTN development can help reduce its related complications. We evaluated the relationship between liver enzymes levels and pre-HTN/HTN in the Azar cohort population.

**Method:**

This cross-sectional study was based on data from the large Azar cohort study and a total of 14,184 participants were included. Pre-HTN and HTN were defined based on the American Heart Association guideline. Serum aspartate aminotransferase (AST), alanine aminotransferase (ALT), alkaline phosphatase (ALP), gamma-glutamyl transferase (GGT) levels were measured by Pars Azmoon kits. The relationship between pre-HTN/HTN and liver enzyme levels was evaluated by logistic regression.

**Results:**

Of 14,184 participants, 5.7% and 39.6% had pre-HTN and HTN, respectively. In the adjusted model, AST levels of 19–23 IU/l were associated with an elevated risk of pre-HTN (OR [95% CI]: 1.24 [1.04–1.48]). A dose-response increase was seen in pre-HTN in relation to ALT, with the highest OR in the third tertile (1.34 [1.09–1.63]). The odds of pre-HTN also increased with GGT in the third tertile (1.25[1.03–1.52]). In addition, the odds of HTN increased with increased levels of AST, ALT, ALP, and GGT, such that the highest ORs were recorded in the third tertile (OR 1.22 [1.09–1.37], 1.51 [1.35–1.70], 1.19 [1.07–1.34], and 1.68 [1.49–1.89], respectively). Among these enzymes, GGT had the highest OR regarding HTN.

**Conclusion:**

This study indicates that AST, ALT, ALP and GGT levels were associated with pre-HTN (except for ALP) and HTN, independent of known risk factors. Hence, it may be possible to use liver enzymes to predict the incidence of pre-HTN and HTN, empowering primary care providers to make the necessary interventions promptly.

## Introduction

Hypertension (HTN) contributes to cardiovascular disease (CVDs) [1], ranking first among 25 factors leading to disability in 2010 [2]. Regarding disability-adjusted life years (DALYs), HTN was the second and first leading risk factor among males and females in 2019, respectively [3]. It also represents a major cause of premature death worldwide [4]. Globally, more than quarter of the world’s population is affected by this condition [4], with a projected rise to an alarming prevalence rate of 29.2% by 2025 [5]. A recent meta-analysis estimated the prevalence of this disease to be 25% in Iran [6]. In developing countries like Iran, population aging secondary to improved screening and management of diseases is likely to increase the overall prevalence of HTN [7, 8]. Non-modifiable risk factors for HTN include gender, age (> 65), and familial history, while modifiable ones include high sodium and low potassium intake, obesity, physical inactivity, stress, and an unhealthy diet [9, 10].

The link between HTN and liver dysfunction has been examined in some studies [11, 12]. Alanine aminotransferase (ALT), aspartate aminotransferase (AST), gamma-glutamyl transferase (GGT), and alkaline phosphatase (ALP) are enzymes that indicate liver function [13], with levels rising in various diseases that affect the liver [14, 15]. Liver enzymes may share an association with elevated blood pressure levels, though the findings in the literature are conflicting. A study in Bangladesh indicated that ALT and GGT are linked with HTN [16], while an Iranian study found no such association [17]. Another surveys by Liu et al. [18] and Park et al. [11] found that elevated AST, ALT, and GGT were associated with incident hypertension. While some researchers have linked higher ALP, ALT, and GGT concentrations with HTN, others have not corroborated this finding. A possible explanation for the association of liver enzymes with HTN may be oxidative stress and inflammation. Inflammatory cytokines by activating the renin-angiotensin system [19], and oxidative stress by causing liver dysfunction contribute to HTN development [20]. Moreover, increased ALP activity might induce vessel calcification and impaired vascular homeostasis, and consequently augment the rate of HTN [21, 22]. Therefore, high level of liver enzymes may be a predictor of pre-HTN and HTN.

As HTN is a disease that has various causes and usually lacks symptoms [23], morbidity and premature mortality can be averted by promptly diagnosing and managing elevated blood pressure levels prior to the development of HTN [24, 25]. Pre-HTN can serve as an early warning for patients and physicians; if individuals are diagnosed in the pre-HTN stage, they can be prevented from developing HTN and its complications. Delineating a link between pre-HTN and liver enzymes may facilitate earlier disease detection. While most prior studies evaluated the relationship between liver enzymes and HTN, studies on liver enzymes’ association with pre-HTN are limited. On the other hand, previous data in the literature are contradictory, and a knowledge gap exists in Iran regarding the study topic. Hence, we assessed the relationship between liver enzyme levels and pre-HTN/HTN in a large sample of adults in Iran, namely the Azar cohort study population.

## Materials and methods

This cross-sectional study used data from the large Azar cohort study to evaluate the association between serum liver enzymes and pre-HTN/HTN. As part of the PERSIAN (Prospective Epidemiological Research Studies in Iran) cohort [26, 27], the Azar cohort study commenced in 2014 and recruited 15,006 (35–70 years old) participants by 2017; it is explained in further detail in prior publications [28]. The protocol of this study was approved by the Ethics Committee of Tabriz University of Medical Sciences (IR.TBZMED.REC.1401.739) and registered in the Research Vice Chancellor of Tabriz University of Medical Sciences, Tabriz, Iran.

All participants were informed of the study procedure. A written informed consent was obtained from all patients or their legal guardians in case of illiteracy. The inclusion criteria for the current study encompassed individuals with pre-HTN and HTN. Participants with non-alcoholic fatty liver (NAFLD), hepatitis B, hepatitis C and missing values were excluded from the present study. Finally, 14,184 subjects remained and statistical analysis was performed on this sample size.

### Data collection

#### Demographic characteristics

Participants’ age, gender, education level, marital status, smoking status, and medical history were recorded using well-designed questionnaires. We measured socioeconomic status using the wealth score index (WSI), dividing the participants into five quintiles using multiple correspondence analysis (MCA). Based on data of physical activity questionnaire, metabolic equivalent of task (METs) was calculated. Smokers were defined as those who continuously used a minimum of one cigarette daily for over half a year, ex-smokers as those who had stopped smoking over a year earlier, and non-smokers were defined as those with no smoking history. ‘Smokers of other tobacco products’ used alternative products like hookahs, water pipes, pipes, or nass.

#### Biochemical analysis

Blood samples were collected at 7:00 to 9:00 AM after overnight fasting (12 to 14 h). We assayed aspartate aminotransferase (AST), alanine aminotransferase (ALT), alkaline phosphatase [13], gamma-glutamyltransferase (GGT) with Pars Azmoon kits. The participants were categorized into the following enzyme level tertiles: for AST ≤ 18, 19–23, and ≥ 24 U/L; for ALT ≤ 18, 19–26, and ≥ 27 U/L; for GGT ≤ 16, 17–25, and ≥ 26 U/L; for ALP ≤ 160, 161–206, and ≥ 207 U/L. In the analysis, each liver enzyme’s first quartile was considered the reference group.

#### Definitions of prehypertension and hypertension

Blood pressure was measured by a trained nurse using a mercury sphygmomanometer (Riester, Germany) twice with 10-minute intervals from both arms separately. The mean of measurements was recorded as the blood pressure of the patient.

In this study, pre-HTN was defined as the systolic blood pressure (SBP) of 120–129 mmHg and the diastolic blood pressure (DBP) < 80 mmHg. HTN was recorded with SBP ≥ 130 mmHg or DBP ≥ 80 mmHg, or a history of HTN [29].

### Statistical analysis

Data were analyzed with SPSS version 11.5 (Chicago, IL, USA). The normality of data was assessed by Skewness and Kurtosis indices and Q-Q plot. We summarized continuous variables with the mean ± standard deviation or median and interquartile range and categorical variables with frequency (percentage). Study groups were compared using the chi-squared, kruskal-Wallis or One-way ANOVA as appropriate. Logistic regression was recruited to analyze the relationship between liver enzyme levels and pre-HTN and HTN (Model 1: unadjusted; Model 2: adjusted for age, gender, socioeconomic status (WSI), MET, diabetes, pre-diabetes, hypertension treatment (if applicable), BMI, waist circumference. The odds ratios (OR) and their 95% confidence intervals (CI) are provided. *P*-values below 0.05 were taken as significant.

## Results

Of 14,184 participants, 802 (5.7%) had pre-HTN, and 5,622 (39.6) had HTN. Table 1 provides a summary of the participants’ characteristics. The prevalence of pre-HTN and HTN was higher among females (*P* < 0.001). The frequency of low education levels was significantly higher in pre-HTN and HTN patients than in apparently healthy participants (*P* < 0.001). Individuals with the poorest WSI were more commonly seen in pre-HTN patients compared to other groups (*P* < 0.001). Moreover, the mean body weight and body mass index (BMI) values were significantly greater in those with HTN (*P* < 0.001). The AST, ALT, GGT, and ALP concentrations were significantly higher in patients with HTN. In addition, the percentage of pre-HTN and HTN patients in the third (highest) tertile of these four liver enzymes was significantly higher than the non-hypertensive population (Table 2).

**Table 1:** General characteristics of the study groups (*n* = 14,184)

Variables	Normal *N* = 7760	Prehypertension *N* = 802	Hypertension *N* = 5622	*P* value
**Gender N (%)**				**<0.001
Male	3724 (48)	397 (49.5)	2278 (40.5)	
Female	4036 (52)	405 (50.5)	3344 (59.5)	
**Education level N (%)**				***<0.001
Illiterate	799 (10.3)	179 (22.3)	1380 (24.6)	
Primary school	2968 (38.3)	324 (40.4)	2262 (40.3)	
Diploma	3148 (40.6)	253 (31.5)	1639 (29.2)	
University	843 (10.9)	46 (5.7)	336 (6.0)	
**Quintiles of wealth index N (%)**				***<0.001
1 (poorest)	1639 (21.1)	223 (27.8)	1471 (26.2)	
2	1161 (15)	150 (18.7)	1067 (19)	
3	1556 (20.1)	170 (21.2)	1177 (20.9)	
4	1814 (23.4)	136 (17)	1007 (17.9)	
5 (richest)	1590 (20.5)	123 (15.3)	900 (16)	
**Marital status N (%)**				**<0.001
Not married	442 (5.7)	66 (8.2)	518 (9.2)	
Married	7318 (94.3)	736 (91.8)	5104 (90.8)	
**Physical activity level (** ^**¶**^ **METs)**				***<0.001
Low	2383 (30.7)	261 (32.5)	2039 (36.3)	
Moderate	2560 (33)	249 (31)	1890 (33.6)	
High	2817 (36.3)	292 (36.4)	1693 (30.1)	
**Current smoking status**				***<0.001
No smoker	5643 (72.7)	598 (74.6)	4501 (80.1)	
Ex-smoker	586 (7.6)	80 (10)	514 (9.1)	
Smoker	1378 (17.8)	109 (13.6)	511 (9.1)	
Smoker other type of tobacco products	153 (2)	15 (1.9)	96 (1.7)	
**Medical History**				
Diabetes	475 (6.1)	106 (13.2)	966 (17.2)	**<0.001
Cardiovascular diseases	177 (2.3)	29 (3.6)	471 (8.4)	**<0.001
	**Mean (SD)**	**Mean (SD)**	**Mean (SD)**	
Age (years)	46.82 ± 8.52	52.62 ± 9.49	52.93 ± 9.06	*<0.001
Height (cm)	163.40 ± 9.34	161.89 ± 9.50	160.98 ± 9.50	*<0.001
Weight (kg)	73.62 ± 12.94	74.97 ± 13.72	78.351 ± 14.07	*<0.001
BMI (kg/m2)	27.60 ± 4.53	28.57 ± 4.57	30.24 ± 4.89	*<0.001
Waist circumference (cm)	91.09 ± 10.72	94.35 ± 10.60	97.74 ± 10.87	*<0.001

P: *One-way ANOVA, **P: Chi-square, *** P: Kruskal-Waliis, BMI; Body mass index

**Table 2:** Comparison of liver enzymes among study groups (*n* = 14,184)

	Normal (*n* = 7760)	Prehypertetnion (*n* = 802)	Hypertension (*n* = 5622)	*P* value
	Mean ± SD	Mean ± SD	Mean ± SD	
**AST (U/L)**	21.74 ± 9.46	21.90 ± 8.60	22.63 ± 10.19	< 0.001
Median (interquartile range)	20 (8)	20 (7)	20 (8)	
**ALT (U/L)**	23.23 ± 12.59	24.35 ± 12.41	25.39 ± 14.87	< 0.001
Median (interquartile range)	20 (11)	22 (11)	22 (13)	
**ALP (U/L)**	182.35 ± 54.94	192.14 ± 54.08	196.56 ± 58.55	< 0.001
Median (interquartile range)	175 (69)	186 (69)	189 (73)	
**GGT (U/L)**	22.43 ± 17.65	25.37 ± 19.69	26.36 ± 20.26	< 0.001
Median (interquartile range)	18 (13)	20 (16)	21 (15)	
**Liver enzymes classification**	**N (%)**	**N (%)**	**N (%)**	
**AST**				< 0.001
1st (≤ 18)	3136 (40.4)	279 (34.8)	2053 (36.5)	
2nd (19–23)	2399 (30.9)	290 (36.2)	1755 (31.2)	
3rd (≥ 24)	2225 (28.7)	233 (29.1)	1814 (32.3)	
**ALT**				< 0.001
1st (≤ 18)	3163 (40.8)	262 (32.7)	1810 (32.2)	
2nd (19–26)	2471 (31.8)	284 (35.4)	1920 (34.2)	
3rd (≥ 27)	2126 (27.4)	256 (31.9)	1892 (33.7)	
**ALP**				< 0.001
1st (≤ 160)	2954 (38.1)	247 (30.8)	1587 (28.2)	
2nd (161–206)	2613 (33.7)	280 (34.9)	1887 (33.6)	
3rd (≥ 207)	2193 (28.3)	275 (34.3)	2148 (38.2)	
**GGT**				< 0.001
1st (≤ 16)	3319 (42.8)	291 (36.3)	1675 (29.8)	
2nd (17–25)	2389 (30.8)	229 (28.6)	1898 (33.8)	
3rd (≥ 26)	2052 (26.4)	282 (35.2)	2049 (36.4)	

P: Kruskal-Wallis, AST; Aspartate aminotransferase, ALT; Alanine aminotransferase, GGT; Gamma-glutamyl transferase, ALP; Alkaline phosphatase

Findings of binary logistic regression indicated that in the adjusted models, AST levels of 19–23 U/L were associated with a higher risk of pre-HTN (1.24; 1.04–1.48) compared to AST levels of ≤ 18 U/L. A dose-response increase was seen in pre-HTN in relation to ALT, with the highest OR in the third tertile (1.34; 1.09–1.63). The odds of pre-HTN also increased with GGT, with the highest OR in the third tertile of the adjusted models (1.25: 1.03–1.52). There was no significant association between the ALP levels and pre-HTN risk (Table 3).

**Table 3:** Association between liver enzymes and pre-hypertension among study groups

Pre-hypertension				
	OR (95%CI)	*P* value	Adjusted OR (95%CI)	*P* value
**AST**				
1st (≤ 18)	Reference		Reference	
2nd (19–23)	1.35(1.14–1.61)	0.001	1.24(1.04–1.48)	0.016
3rd (≥ 24)	1.17(0.98–1.41)	0.080	1.08(0.89–1.31)	0.413
**ALT**				
1st (≤ 18)	Reference		Reference	
2nd (19–26)	1.38(1.16–1.65)	< 0.001	1.19(0.99–1.43)	0.057
3rd (≥ 27)	1.45(1.21–1.74)	< 0.001	1.34(1.09–1.63)	0.004
**ALP**				
1st (≤ 160)	Reference		Reference	
2nd (161–206)	1.28(1.07–1.53)	0.007	1.08(0.90–1.31)	0.367
3rd (≥ 207)	1.50(1.25–1.79)	< 0.001	1.05(0.87–1.27)	0.57
**GGT**				
1st (≤ 16)	Reference		Reference	
2nd (17–25)	1.09(0.91–1.31)	0.334	0.92(0.76–1.12)	0.438
3rd (≥ 26)	1.56(1.31–1.86)	< 0.001	1.25(1.03–1.52)	0.024

Adjusted for age, gender, WSI (socioeconomic status), Physical Activity (MET), BMI, waist circumference, diabetes, prediabetes

Aspartate aminotransferase, ALT: Alanine aminotransferase, GGT: Gamma-glutamyl transferase, ALP: Alkaline phosphatase

Binary logistic regression revealed that the odds of HTN significantly increased with elevated levels of AST, ALT, ALP, and GGT, with the maximum ORs recorded in the highest tertile of each enzyme. These OR values were 1.22 (1.09–1.37), 1.51 (1.35–1.70), 1.19 (1.07–1.34), and 1.68 (1.49–1.89), respectively (Table 4).

**Table 4:** Association between liver enzymes and hypertension among study groups

Hypertension				
	OR (95%CI)	*P* value	Adjusted OR (95%CI)	*P* value
**AST**				
1st (≤ 18)	Reference		Reference	
2nd (19–23)	1.11(1.02–1.21)	0.009	1.09(0.98–1.21)	0.106
3rd (≥ 24)	1.24(1.14–1.35)	< 0.001	1.22(1.09–1.37)	< 0.001
**ALT**				
1st (≤ 18)	Reference		Reference	
2nd (19–26)	1.35(1.25–1.47)	< 0.001	1.14(1.02–1.28)	0.015
3rd (≥ 27)	1.55(1.42–1.69)	< 0.001	1.51(1.35–1.70)	< 0.001
**ALP**				
1st (≤ 160)	Reference		Reference	
2nd (161–206)	1.34(1.23–1.46)	< 0.001	1.08(0.97–1.21)	0.14
3rd (≥ 207)	1.82(1.67–1.98)	< 0.001	1.19(1.07–1.34)	0.001
**GGT**				
1st (≤ 16)	Reference		Reference	
2nd (17–25)	1.57(1.44–1.71)	< 0.001	1.37(1.22–1.54)	< 0.001
3rd (≥ 26)	1.97(1.81–2.15)	< 0.001	1.68(1.49–1.89)	< 0.001

Adjusted for age, gender, BMI, WSI (socioeconomic status), Physical activity (MET), and hypertension treatments, BMI, waist circumference, Diabetes, Prediabetes

Aspartate aminotransferase, ALT; Alanine aminotransferase, GGT; Gamma-glutamyl transferase, ALP; Alkaline phosphatase

## Discussion

The prevalence of pre-HTN and HTN is increasing globally [30]. Early diagnosis of pre-HTN and prompt management to prevent HTN development is critical. Previous epidemiological studies reported inconsistent findings concerning the link between different liver enzymes and pre-HTN or HTN [31, 32]. Finding a clear association between liver enzymes and pre-HTN or HTN could help predict these conditions. To our knowledge, our work is the most extensive population-based study to examine the association of serum ALT, AST, GGT, and ALP levels and pre-HTN and HTN. Our findings revealed that these enzymes had significantly higher serum concentrations in pre-HTN and HTN patients than in non-hypertensive Iranian Azar cohort study participants. Moreover, a significant association existed between increased enzyme levels and elevated odds of pre-HTN (except for ALP) and HTN, even after adjusting for age, gender, WSI, BMI, MET, waist circumference, diabetes, prediabetes, and HTN treatment (if applicable).

Similar to our findings, the Tehran Lipid and Glucose Study reported that the elevated ALT, GGT, and ALP serum concentrations were associated with HTN [12]. Another cohort study by Khalili et al. in Iran demonstrated that increased serum ALP activity was associated with increased odds of HTN, however, there was no significant association between ALT, AST, and GGT with HTN [17]. Tahmasebi-Fard et al. showed that the serum GGT level offers some value in promptly detecting HTN [33], and Kohsari et al. found that liver enzyme levels could be considered for the early diagnosis of HTN [34]. Sakboonyarat et al. linked HTN with elevated odds of higher AST and ALT levels than optimal blood pressure in Thailand [35]. Similarly, Rahman et al. demonstrated a higher rate of abnormal liver enzyme levels in people with HTN, linking ALT and GGT with HTN among Bangladeshi adults [16]. Some prior investigations have also suggested an association between elevated serum ALP and an increased risk of HTN [36, 37]. Other surveys reported no significant association between some of serum AST, ALT, and GGT levels and odds of HTN [17, 38]. This discrepancy might be due to differences in age, gender, region, cut-offs for HTN detection, adjusted confounders in the analyses, and unmeasured confounders.

The link between liver enzymes and pre-HTN, however, has sparsely been studied. NAFLD is a risk factor for pre-HTN incidence [39] and the progression of pre-HTN to HTN [19]. Similar to our findings, Zhu et al. associated elevated GGT levels with pre-HTN [40]. Moreover, Qin et al. used multiple logistic regression analyses to show a significant association between GGT levels and pre-HTN [41].

The pathophysiological processes that explain the association between higher liver enzyme levels and an elevated risk of pre-HTN/HTN remain elusive, though prior studies make some suggestions. NAFLD occurs more in HTN patients than in non-hypertensive individuals [42, 43]. It is well-documented that HTN is associated with hyperinsulinemia and metabolic syndrome, leading to fatty liver disease [44, 45]. Elevated AST and ALT levels appear to be the predominant laboratory-based markers associated with such abnormalities [45]. Increased bone-type ALP activity might induce vessel calcification and impair vascular homeostasis, thereby augmenting the rate of HTN [21, 22]. Furthermore, elevated ALP concentrations might also potentiate atherosclerosis development [46].

According to animal studies, angiotensin II activates liver stellate cells during the development of hepatic fibrosis [47, 48]. Furthermore, a clinical study established that an elevated angiotensin II level could independently predict NAFLD [49]. NAFLD promotes systemic inflammation via damage-associated molecular patterns [50] and altered hepatokine profiles [51]. Pro-inflammatory cytokines like tumor necrosis factor-alpha and interleukin-6 activate the renin-angiotensin system and contribute to HTN development [52, 53].

An alternative link between HTN and increased liver enzyme concentrations may be oxidative stress, which is pivotal in HTN development [20]. Polymorphisms in the glutathione-S transferase gene, as an antioxidant enzyme gene, may increase an adult’s HTN risk [54] while also inducing hepatocellular injuries [55]. In confirmation of this mechanism, some studies have shown that cellular GGT is involved in the catabolism of glutathione [49, 56] and the production of reactive oxygen species [57]. Additionally, NAFLD could affect vascular constriction and dilation [58]. These patients have impaired endothelial nitric oxide synthase function [59] and accumulate asymmetrical dimethylarginine, an endogenous nitric oxide synthase inhibitor [60], causing diastolic dysfunction. A bioinformatic analysis revealed that NAFLD and HTN share several biologic pathways, including antioxidant activity, lipid binding, myeloid leukocyte activation, and disease-associated genes [61]. These findings support an association between NAFLD and HTN. While in the present study the participants with NAFLD were excluded, there might be subjects with NAFLD whose disease had not been diagnosed yet.

### Strengths and limitations

Our study had some strengths and limitations. As part of a prospective population-based cohort study, this cross-sectional investigation had a relatively large sample size and considered the potential confounders when assessing the link between liver enzymes and pre-HTN/HTN. However, the cross-sectional design restrains the interpretation of cause-and-effect relations.

## Conclusion

The present study found significantly elevated ALT, AST, GGT, and ALP concentrations in pre-HTN and HTN patients compared with non-hypertensive participants. Moreover, elevations in these enzymes were associated with increased odds of pre-HTN (except for ALP) and HTN. Therefore, these liver enzymes can help primary care physicians as early indicators of pre-HTN and HTN. Nevertheless, further investigations are needed to confirm our findings in other age groups and populations. In addition, prospective, large-scale studies with longer follow-up times are required to delineate the underlying mechanisms that link elevated liver enzymes with the development of pre-HTN and HTN in the general population.

## Data Availability

The data that support the findings of this study are available from the corresponding author, upon reasonable request and with permission of [Vice Chancellor for Research of Tabriz University of Medical Sciences]. The data are not publicly available due to their containing information that could compromise the privacy of research participants.
